# Association between heavy metals and colon cancer: an ecological study based on geographical information systems in North-Eastern Iran

**DOI:** 10.1186/s12885-021-08148-1

**Published:** 2021-04-15

**Authors:** Behzad Kiani, Fatemeh Hashemi Amin, Nasser Bagheri, Robert Bergquist, Ali Akbar Mohammadi, Mahmood Yousefi, Hossein Faraji, Gholamreza Roshandel, Somayeh Beirami, Hadi Rahimzadeh, Benyamin Hoseini

**Affiliations:** 1 Department of Medical Informatics, School of Medicine, Mashhad University of Medical Sciences, Mashhad, Iran; 2 Visualization and Decision Analytics (VIDEA) lab, Centre for Mental Health Research, Research School of Population Health, College of Health and Medicine, The Australian National University, Canberra, Australia; 3 Ingerod, Brastad, Sweden (formerly with the UNICEF/UNDP/World Bank/WHO Special Programme for Research and Training in Tropical Diseases, World Health Organization), Geneva, Switzerland; 4 Department of Environmental Health Engineering, Neyshabur University of Medical Sciences, Neyshabur, Iran; 5 Department of Environmental Health Engineering, School of Public Health, Iran University of Medical Sciences, Tehran, Iran; 6 Department of Environmental Health Engineering, Health Center, Babol University of Medical Sciences, Babol, Iran; 7 Golestan Research Center of Gastroenterology and Hepatology, Golestan University of Medical Sciences, Gorgan, Iran; 8 Department of Environmental Health Engineering, Faculty of Health and Environmental Health Research Center, Golestan University of Medical Sciences, Gorgan, Iran; 9 Pharmaceutical Research Center, Mashhad University of Medical Sciences, Mashhad, Iran; 10 Department of Health Information Technology, Neyshabur University of Medical Sciences, Neyshabur, Iran

**Keywords:** Colon cancer, Geographical information systems, Golestan, Heavy metals, Iran, Ordinary least square, Regression model, Spatial analysis, Trace elements

## Abstract

**Background:**

Colorectal cancer has increased in Middle Eastern countries and exposure to environmental pollutants such as heavy metals has been implicated. However, data linking them to this disease are generally lacking. This study aimed to explore the spatial pattern of age-standardized incidence rate (ASR) of colon cancer and its potential association with the exposure level of the amount of heavy metals existing in rice produced in north-eastern Iran.

**Methods:**

Cancer data were drawn from the Iranian population-based cancer registry of Golestan Province, north-eastern Iran. Samples of 69 rice milling factories were analysed for the concentration levels of cadmium, nickel, cobalt, copper, selenium, lead and zinc. The inverse distance weighting (IDW) algorithm was used to interpolate the concentration of this kind of heavy metals on the surface of the study area. Exploratory regression analysis was conducted to build ordinary least squares (OLS) models including every possible combination of the candidate explanatory variables and chose the most useful ones to show the association between heavy metals and the ASR of colon cancer.

**Results:**

The highest concentrations of heavy metals were found in the central part of the province and particularly counties with higher amount of cobalt were shown to be associated with higher ASR of men with colon cancer. In contrast, selenium concentrations were higher in areas with lower ASR of colon cancer in men. A significant regression equation for men with colon cancer was found (F(4,137) = 38.304, *P* < .000) with an adjusted R^2^ of 0.77. The predicted ASR of men colon cancer was − 58.36 with the coefficients for cobalt = 120.33; cadmium = 80.60; selenium = − 6.07; nickel = − 3.09; and zinc = − 0.41. The association of copper and lead with colon cancer in men was not significant. We did not find a significant outcome for colon cancer in women.

**Conclusion:**

Increased amounts of heavy metals in consumed rice may impact colon cancer incidence, both positively and negatively. While there were indications of an association between high cobalt concentrations and an increased risk for colon cancer, we found that high selenium concentrations might instead decrease the risk. Further investigations are needed to clarify if there are ecological or other reasons for these discrepancies. Regular monitoring of the amount of heavy metals in consumed rice is recommended.

## Introduction

With around 18 million new cases and 9 million deaths annually [1], colon cancer ranks the third most frequent malignancy in the world and the fourth most common in Iran [2, 3]. These figures may be skewed in the direction of a higher percentage in low-income countries, and the scientific literature reveals that many causes can be involved in its development [4–8]. Some factors (obesity, physical inactivity, diet, smoking, and access to medical services) are modifiable [5, 8], while others (age, gender, family history and genetics) are not [4, 6, 7]. Exposure to environmental pollutants, such as heavy metals, constitutes a potential risk that falls somewhat between these two categories [9–12].

Heavy metals, defined as such due to their relatively high atomic weights (and thus also densities), constitute one of the main environmental pollutants that can cause critical problem for all organisms [13–16]. They exist in water, air, soil and consequently also in food making it essential to assess the amount of toxic trace elements in these compartments with particular emphasis on the food chain. Cadmium, lead, arsenic and mercury are generally toxic to soil microbial populations, plants as well as humans [14, 17, 18], while copper, nickel and zinc act as micronutrients in low concentrations but can still be toxic when contents are high [19, 20]. They enter the food chain when accumulated in plant tissues resulting in further accumulation in human organs over time [21] that eventually can cause adverse health effects [22]. Soil, growth media, pesticides, fertilizers and nutrient solutions are the main sources of these trace elements in plants [23]. Rice can be harmful if the soil where it is produced is contaminated by these metals [17, 24] and this amounts to a potentially major worldwide problem as rice is a common staple food.

Many recent studies have highlighted the association between heavy metals and some forms of cancer [25–29]. For example, Adimalla et al. [25] investigated the association between heavy metals in soil and health risks for adults and children in India, and found high concentration of arsenic and chromium which are potentially associated with increased cancer risk for both adults and children; Fei et al. [26] assessed the association between food contamination of heavy metals and the incidence and spatial distribution of stomach cancer in Hangzhou, China finding a significant association between multiple heavy metals and with stomach cancer risk, despite the fact that each metal contamination on its own did not reach statistical significance; and Sohrabi et al. [28] found evidence for involvement of heavy metals in the development of colorectal cancer based on a cross-sectional study of tissue levels of trace elements performed in Tehran, the capital of Iran. On the other hand, a 20-year old study in the United States on the potential association between the level of lead in the blood and cancer mortality in general, found no significant association between lead levels and increased risks of cancer mortality [27].

Focussing on rice and the potential cancer risks related heavy metal contamination, Kukusamude et al. [30] recently measured the contents of chromium, zinc, nickel, copper, manganese, cobalt, arsenic and cadmium in 55 Thai local rice products. Assessing the potential impact of this on Thai populations with lifetime consumption of locally produced rice, they came up with risks of developing cancer varying from 5 to 30 per 10,000 population; Al-Saleh et al. [31] determined the levels of lead, cadmium, methyl mercury and arsenic in 37 brands of imported rice commonly consumed in Saudi Arabia and found that long-term consumption of rice contaminated with heavy metals, particularly arsenic, pose potential cancer risks; and Rahimzadeh et al. (2017) measured the level of heavy metals in rice harvested in the Golestan Province, Iran, revealing that these elements in rice may act as possible risk factors for oesophagus cancer.

People living close to each other should have more similar exposure to heavy metals in food. Spatial analysis could help scientists to explore how heavy metals and colon cancer incidence rates are associated. Such studies are conducted by Geographical Information Systems (GIS) [32], which consider the geographical distribution patterns of climate, diseases, contaminants, etc. and based on such data establish statically significant links of potential hazard. Ordinary least square (OLS) is a practical approach for estimating the relationship between a response variable and explanatory variables [33]. Using OLS in a study in north-eastern Iran to explore the relationship between colorectal cancer incidence and various explanatory variables, including Body Mass Index (BMI), daily fibre intake and red meat consumption, Goshayeshi et al. [34] found that these explanatory variables, except red meat consumption, were associated with colorectal cancer incidence in some way.

Colorectal cancer has increased in Iran and elsewhere [1–3]. Although many studies indicate trace elements, such as heavy metals [9–12], there is still a lack of data to unequivocally link consumption of heavy metals and colorectal cancer. In search of aetiology, there is a need to investigate how strong this association is. Since the majority of populations in Iran are stationary and also regularly consume rice, which generally is their main meal, we felt that it would be useful to measure the association between colon cancer and locally grown rice in areas known to be contaminated by heavy metals.

## Methods

### Setting

**Fig. 1:**
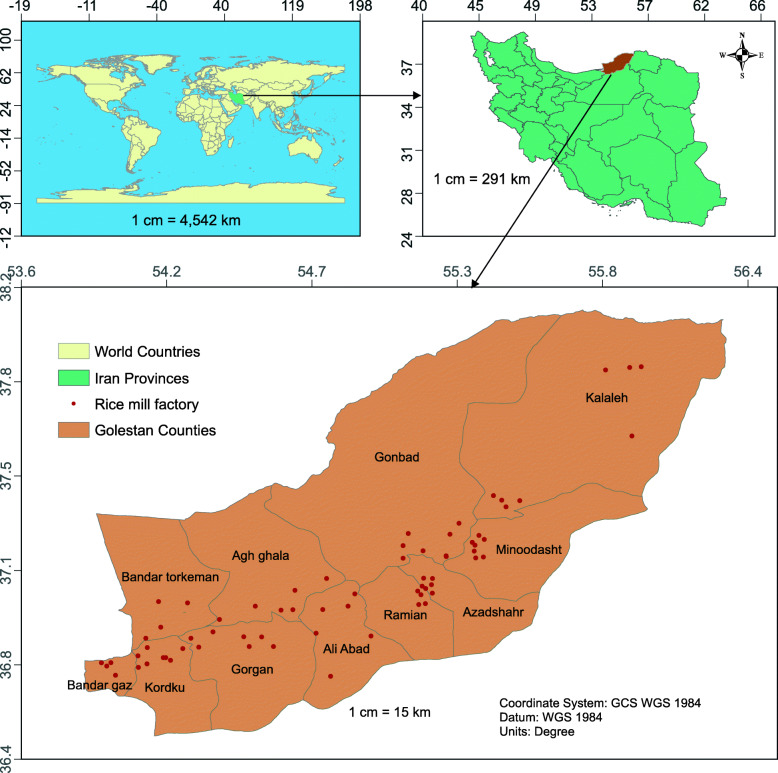
Distribution of milling factories in the study area in 2018

This study was conducted in 11 counties of the province of Golestan in Iran in 2018, which covers an area of 20,367 km^2^ with a population of 1,868,819 people and includes also 69 of the 90 milling factories in the province (Fig. 1).

### Data sources

We used two different data sources: 1) Iranian Golestan Population-based cancer registry to extract individuals with colon cancer; and 2) polarograph assessment of the amount of heavy metals in rice samples including cadmium (Cd), cobalt (Co) copper (Cu), lead (Pb), nickel (Ni), selenium (Se) and zinc (Zn). We calculated the age-standardized rate (ASR) of colon cancer cases and used it as the dependent variable for statistical modelling.

### Sampling

The number of rice milling factories required for the study was estimated based on a study conducted by Zazouli et al. [35] on cultured rice in the province of Mazandaran, whose climate is similar to that of Golestan. As lead comprised a large proportion of heavy metals in our testing, the number of factories (sample size) was calculated based on this metal. Using the formula below, the confidence interval level (α = 0.05) and error rate were assumed not to exceed 0.05%.

1 $$ N=\left({\mathrm{z}}_{\left(1-\alpha /2\right)}^2\ast {\sigma}^2\right)/ {d}^2 $$

where N is number of factories, z the value of the normal variable with confidence level α; σ the standard deviation (SD) of the amount of lead; and d the error rate.

The required number of milling factories was determined to be 62. Considering the adequate allocation of the area under cultivation, for some counties the sample size was estimated at 1 or 2, which was very small. As a result, at least 4 cases were considered for each county and ultimately, a total of 69 rice milling factories were investigated in this study. The number of samples for each county in the study area is shown in Table 1.

**Table 1:** The number of samples in the study area

County name	Number of rice milling factories	The area under cultivation (In acres)	Required sample size
Bandar gaz	6	2650	4
Kordku	8	6408	8
Bandar torkeman	4	515	4
Agh ghala	6	4543	6
Gorgan	18	5209	7
Ali Abad	7	6200	6
Azadshahr	12	5275	7
Gonbad	8	6530	8
Ramian	6	1819	4
Minoodasht	11	4985	7
Kalaleh	4	970	4
Total	90	45,714	69

Directly after delivery of harvested rise to the milling factories from the fields, rice samples were drawn from the factories in each county. The plastic bags were transported to the environmental chemistry laboratory of the school of public health in Gorgan and kept in the refrigerator until analysis. All seven heavy metals were measured in each sample.

#### Visualising the spatial pattern of heavy metals

We used spatial interpolation to visualise the spatial pattern of heavy metals across the study area through the generation of a so-called heat map based on GIS software where the quantitative value of each metal is represented by pixels in a computer’s raster layer. This approach predicts the values of unobserved areas based on known point values, thereby creating a “statistical surface”. There are different spatial interpolation methods and the most important issue is to generate a map as accurate as possible, i.e. based on the method with the least amount of error for the specific purpose at hand [36]. In our case, we performed the interpolation by inverse distance weighting (IDW) [37], Spline [38] and Kriging [34]. After that, we evaluated the three methods by measuring the difference between the predicted and the observed amount of heavy metals in points where the observed values were available, i.e. at the location of the rice milling factories. The average square error (R^2^) was measured and the method with the lowest amount of error was chosen for the visualisation.

### Exploratory regression mining

We used this approach to develop several regression models. Here, the explanatory variables comprised the spatial presence of the heavy metals, and the dependent one was the ASR of men and women with colon cancer. We used ArcGIS 10.5 (ESRI, Redlands, CA, USA) to conduct the exploratory regression analyses. This tool runs many ordinary least square (OLS) models using all possible combinations for a list of independent variables and evaluates which model has the best fit. According to ESRI [39], The six items addressed in assessing an appropriate regression model are:

1. The expected coefficient signs: they should be consistent with the scientific literature, e.g., in recent literature lead has had a positive correlation with cancer occurrence and we expected to find a consistent result in our study.
2. Lack of redundancy: the variance inflation factor (VIF) should be smaller than 7.5 for any variable, otherwise there might be collinearity between dependent exposure variables.
3. Significance of coefficients: Probability and Robust Probability in the result should be checked to assess if coefficients are statistically significant.
4. Normal distribution of residuals: the sum of the residuals should be zero with a SD of 1 and a statistically significant Jarque-Bera test [40] must be avoided.
5. Strong adjusted R^2^: this value must be > 0.5.
6. Lack of spatial autocorrelation among OLS residuals: the Global Moran’s *I* [41] is used to confirm this.

## Results

Overall, 1184 colon cancer cases (656 males vs. 528 females) were identified in Golestan Province, Iran in 2018. The ASR ranged between 3.1 and 18.6 for males among the counties with Ramian having the lowest rate and Gorgan the highest. For the females, it ranged between 1.4 and 14, Minodasht having the lowest rate and Gorgan again the highest.

The average concentration and ranges for each of the seven heavy metals investigated are shown in Table 2. Gonbad had unusually high concentrations of all the heavy metals, except zinc and selenium.

**Table 2:** The average amount of heavy metals in the counties of Golestan province, Iran, in 2018

County Name	Zn (mg/kg)	Cd (mg/kg)	Pb (mg/kg)	Cu (mg/kg)	Ni (mg/kg)	Co (mg/kg)	Se (mg/kg)
Bandar Torkeman	26.357	0	0	1.0182	0.842	0	2.96
Kordku	25.579	0.0155	0.1385	1.2265	2.8085	0.00675	1.4234
Bandar Gaz	32.4563	0	1.46075	1.7875	3.90038	0.102625	2.538
Agh Ghala	25.6238	0	0.196833	2.3977	0.6455	0	2.73016
Gorgan	31.3155	0.007643	1.1145	3.0515	1.3993	0.0185	1.1963
Ali Abad	20.3349	0.0085	1.7211	0.7619	3.3621	0	2.3765
Kalaleh	26.140125	0	0.28	3.028	1.290771	0.1005	4.7585
Gonbad	38.7784688	0.101688	2.235375	6.993281	4.417125	0.110625	3.587375
Minoodasht	54.7726429	0	1.618571	5.633857	2.283243	0	0.494614
Azadshahr	56.1412857	0.073786	0.7835	10.1745	2.577571	0	1.431738
Ramian	45.648	0	1.23575	7.62775	0.954	0	2.3255
**Range**	20.3349–56.1412857	0–0.101688	0–2.235375	0.7619–10.1745	0.6455–4.417125	0–0.110625	0.494614–4.7585

*mg* milligrams, *kg* kilograms

**Fig. 2:**
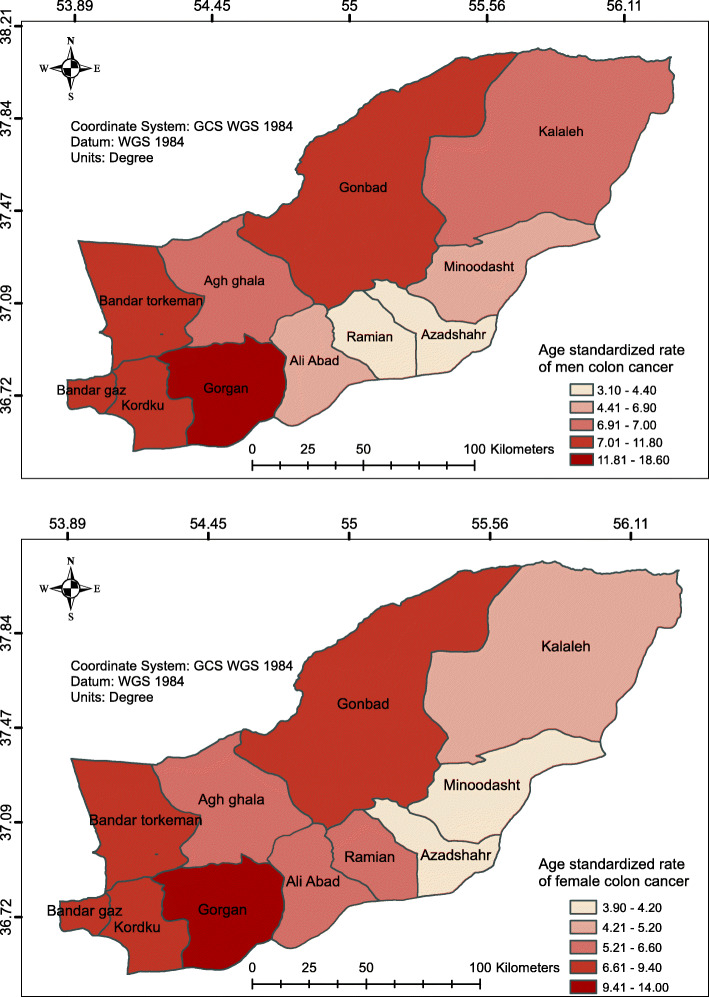
The geographical distribution of age-standardized colon cancer rate in the study area

The geographical distribution of the ASR of colon cancer is shown in Fig. 2. Gorgan County demonstrates the highest ASR, while Azadshahr and Ramian were at the lower end of the range.

**Fig. 3:**
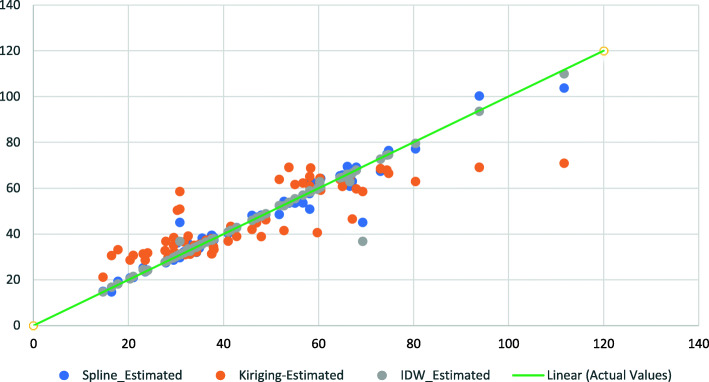
Comparison of different methods to interpolate the amount of heavy metals

Figure 3 shows the residuals and the differences between observed and predicted values for the different spatial interpolation methods. We found the IDW method resulting in the lowest amount of error.

**Fig. 4:**
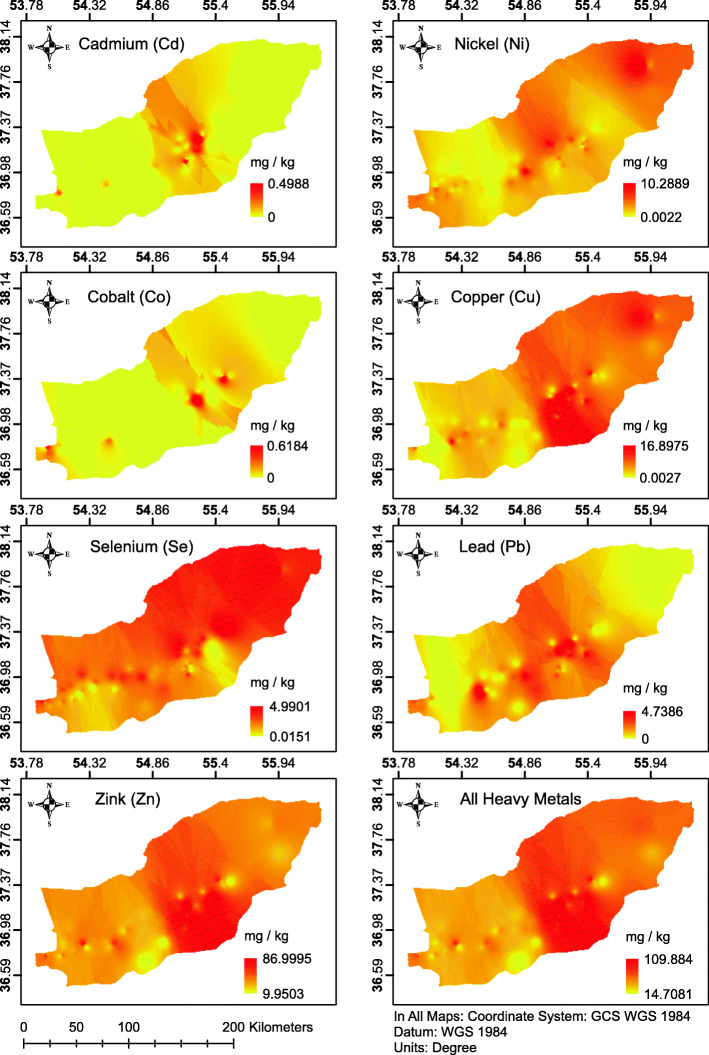
Concentrations of the different heavy metals in the study as expressed as heat maps

Figure 4 shows the spatial distribution of explanatory variables used to build the regression model to predict the ASR of colon cancer with results expressed as a set of heat maps, one for each metal. The figure reveals a high level of heavy metal concentration in the central part of the study area. However, nickel and selenium had a higher concentration in the North-east of the study area.

The exploratory regression model was run for men and women separately, considering seven independent variables. Among these variables, selenium and cobalt were significant in 20.3% and 12.5% of the created models for men with colon cancer, respectively. However, lead was not significant in any model and only selenium and cobalt had a clear direction in all models. In fact, selenium had an inverse association with ASR of colon cancer in men, while cobalt had a positive association with the dependent variable in all models. We did not find a significant outcome for colon cancer in women. Tables 3 and 4 include the sign and significance level of the independent variables in all regression models for men and women with colon cancer, respectively.

**Table 3:** Summary of variables significance for men colon cancer

Variable	Significant [%]	Negative [%]	Positive [%]	Coefficient Variable for model 1	Coefficient Variable for model 2
SE	20.31	100.00	0.00	−6.069037	−4.614778
CO	12.50	0.00	100.00	120.329780	103.938969
ZN	9.38	71.88	28.12	−0.405843	–
NI	9.38	51.56	48.44	−3.086802	−3.537392
CD	6.25	25.00	75.00	80.603505	132.584633
CU	3.12	87.50	12.50	–	−1.699960
PB	0.00	39.06	60.94	–	–

**Table 4:** Summary of variables significance for women colon cancer

Variable	Significant [%]	Negative [%]	Positive [%]	Coefficient Variable for model 1	Coefficient Variable for model 2
ZN	3.12	93.75	6.25	−0.181621	−0.170447
CO	1.56	4.69	95.31	55.023949	29.755027
SE	1.56	100.00	0.00	−2.558817	−1.807687
CD	0.00	35.94	64.06	–	–
PB	0.00	21.88	78.12	–	–
CU	0.00	81.25	18.75	–	–
NI	0.00	79.69	20.31	−0.991396	–

Two models for men (Model 1 and Model 2) were identified using the Exploratory Regression, which fit all the six requirements mentioned in the method section. As Table 5 shows, their adjusted *R*^2^ values were above 0.7, which illustrated the high performance of the models, and they explored five significant variables (*p*-value < 0.05). Model 1 showed a better fit with higher adjusted *R*^2^ of 0.77 and lower Akaike’s Information Criterion (AICc) of 89.14 for men with colon cancer (Table 5).

**Table 5:** Highest Adjusted R^2^ Results for male colon cancer

id	Exploratory Regression Model					AdjR^2^	AICc	JB	K (BP)	VIF	SA
1	-ZN***	+CD**	-NI**	+CO***	-SE***	0.769988	89.139950	0.994852	0.300309	4.262827	0.544661
2	-CU***	+CD**	-NI**	+CO**	-SE***	0.722625	91.199583	0.833018	0.416308	3.715638	0.308703

*AdjR*^*2*^ Adjusted R-Squared, *AICc* Corrected Akaike’s Information Criterion, *JB* Jarque-Bera *p*-value, *K (BP)* Koenker (BP) Statistic *p*-value, *VIF* Max Variance Inflation Factor; SA: Global Moran’s I *p*-value; Model Variable sign: (+/−); Variable significance (* = 0.10; ** = 0.05; *** = 0.01)

None of models met all six requirements mentioned in the method section for women with colon cancer. However, Table 6 shows the two best models (Model 1 and Model 2) with the highest adjusted *R*^2^ and lowest AICc.

**Table 6:** Highest Adjusted R^2^ Results for female colon cancer

id	Exploratory Regression Model					AdjR^2^	AICc	JB	K (BP)	VIF	SA
1	-ZN**	-NI*	+CO**	-SE**	–	0.31	93.09	0.59	0.10	4.26	0.89
2	-ZN**	+CO*	-SE*	–	–	0.30	127.32	0.81	0.43	4.66	0.48

*AdjR*^*2*^ Adjusted R-Squared, *AICc* Corrected Akaike’s Information Criterion, *JB* Jarque-Bera p-value, *K (BP)* Koenker (BP) Statistic p-value, *VIF*: Max Variance Inflation Factor; SA: Global Moran’s I *p*-value; Model Variable sign: (+/−); Variable significance (* = 0.10; ** = 0.05; *** = 0.01)

## Discussion

To our knowledge, this is the first study in Golestan Province, Iran to identify the association of the spatial patterns of ASR of colon cancer with exposure to heavy metals in rice. Our findings show that the exposure level of cobalt was positively associated with ASR of men with colon cancer at the county level, while higher exposure level of selenium was associated with lower ASR of colon cancer in this group. However, the study did not find any significant association between the exposure level of the heavy metals and ASR of colon cancer in women residing in a county. These findings are in line with previous studies [42–46]. While a high concentration of the heavy metal distribution was observed in the central part of the study area, nickel and selenium had a higher concentration in the North-east. There is no specific industry in these areas, so the high levels of these two metals in these regions may depend on the soil’s natural mineral contents.

Although selenium is a micronutrient required for the functioning of a number of enzymes in both humans and animals, it may cause adverse health effects in high concentration [15]. A study by Rahimzadeh et al. [47] in Golestan Province reported that the selenium concentration in high-risk areas of oesophageal cancer was significantly higher than in low-risk areas. The results of our study are in line with two previous meta-analyses [42, 43] that revealed a reverse relationship for colon cancer incidence in these areas, at least for men. Another study [46] found that lower concentrations of selenium with thresholds of 55 μg/l and 65 μg/l in Poland and Estonia, respectively, were associated with a higher risk of colorectal cancer. According to Fernandez-Banares et al. [48], a high level of selenium (≥82.11 μg/L) decreases the risk of colorectal adenomas for those aged < 60 years. Although they found no significant association for this in groups aged ≥60 years, another study [44] reported a low risk of colorectal adenomas for those aged ≥67 years in areas with a high level of selenium. Our study is also in line with the study by Peters et al. [44] that did not see any significant association between selenium concentrations and colon cancer in women. It is recommended that the impact of age and gender be further considered within assessing the association between selenium concentration and risk of colon cancer, which thus remains for future studies.

As a part of vitamin B-12, cobalt is beneficial. However, excessive concentration of cobalt may damage human health [49]. Previous studies investigated the association between exposure levels of the cobalt and cancer incidence and reported controversial findings [50–52]. While some of these studies [50, 51] reported that exposure level of the cobalt might increase the risk of cancer of the upper gastrointestinal tract and lungs, Sauni et al. [52] suggest that occupational-exposure to cobalt may not be associated with an increased overall cancer risk. Our study revealed that exposure level of cobalt is significantly associated with colon cancer incidence in men. Contaminated soil and water may be the source of environmental exposure to heavy metals [53], and contamination of the agricultural soil and water with these elements has been reported previously in some areas of Iran [54]. Further studies are needed to find the source of the high level of cobalt concentration in Golestan, especially in the central region of this province. Further, we need to conduct future studies to assess the association between cobalt exposure levels and colon cancer incidence at the individual level.

The environmental presence of heavy metals found in this study and also by many other authors [55–58] may explain spatial variation in colon cancer pattern. Lead, for example, is becoming known as of major health concern, and the exposure level of this element may enhance the cancer risk in general [55, 56]. A study by Halimi et al. [59] performed in Hamedan Province, Iran, strongly brought forth the hypothesis that exposure level of heavy metals, especially lead, might result in a high incidence of colorectal cancer. Although our study reinforced their hypothesis regarding selenium and cobalt, the results could not confirm that exposure level of lead enhances the risk of colon cancer.

Genetically inherited autosomal disorders, such as the Lynch syndrome, increases the risk of colon cancer [60], and it is estimated 10–14% of cases to be at a high risk of this type of cancer in Iran [6, 61–63]. However, we found no study addressing this subject in Golestan Province. As the high ASR of colon cancer in some regions of the study area, such as Gonbad and Gorgan, may be due to a high prevalence of Lynch syndrome, the screening for this disorder among patients with colon cancers is strongly suggested.

### Limitation(s)

The population census in Iran is carried out every 5 years, so we used census data of 2015 for this research because we did not have an annual census data.

## Conclusion

The GIS-based approach used in this study introduced an opportunity to assess the geographical distribution of different cancers in relation to environmental risk factors. The results of this first study in Iran to ecologically investigate the potential association between colon cancer and locally grown rice in areas contaminated by heavy metals indicate that it is likely that increased exposure to heavy metals in consumed rice could impact the colon cancer incidence. The inverse association between high selenium concentrations and colon cancer incidence in men reinforces previous findings indicating that this may decrease the risk, while there might be an association between high cobalt concentrations and increased risk for colon cancer. Although further studies beyond the ecological approach applied here are needed to confirm these findings, regular monitoring of the amount of heavy metals in consumed rice is recommended.

## Data Availability

Data will be provided upon request with the permission of the corresponding authors.

## Abbreviations

OLS model: Ordinary Least Squares model
AdjR2: Adjusted R-Squared
AICc: Akaike’s Information Criterion
JB: Jarque-Bera *p*-value
K (BP): Koenker’s studentized Breusch-Pagan p-value
VIF Maximum: Variance Inflation Factor
SA Global Moran’s I p-value: a measure of residual spatial autocorrelation
Model Variable direction: (+/−)
Model Variable significance: (* = 0.10, ** = 0.05, *** = 0.01).
